# Role of Bcl-xL/Beclin-1 in synergistic apoptotic effects of secretory TRAIL-armed adenovirus in combination with mitomycin C and hyperthermia on colon cancer cells

**DOI:** 10.1007/s10495-014-1028-6

**Published:** 2014-08-26

**Authors:** Seog-Young Kim, Dae-Hee Lee, Xinxin Song, David L. Bartlett, Yong Tae Kwon, Yong J. Lee

**Affiliations:** 1 Department of Surgery, School of Medicine, University of Pittsburgh, Hillman Cancer Center, 5117 Centre Ave. Room 1.46C, Pittsburgh, PA 15213 USA; 2 Department of Pharmacology & Chemical Biology, School of Medicine, University of Pittsburgh, Hillman Cancer Center, 5117 Centre Ave. Room 1.46C, Pittsburgh, PA 15213, USA; 3 Protein Metabolism Medical Research Center and Department of Biomedical Science, College of Medicine, Seoul National University, Seoul, 110-799 Korea

**Keywords:** Mitomycin C, Ad.TRAIL, Hyperthermia, Mitochondria-dependent pathway, Bcl-xL, Beclin-1

## Abstract

In this study, we attempted to develop a multimodality approach using chemotherapeutic agent mitomycin C, biologic agent tumor necrosis factor-related apoptosis-inducing ligand (TRAIL/Apo-2L), and mild hyperthermia to treat colon cancer. For this study, human colon cancer LS174T, LS180, HCT116 and CX-1 cells were infected with secretory TRAIL-armed adenovirus (Ad.TRAIL) and treated with chemotherapeutic agent mitomycin C and hyperthermia. The combinatorial treatment caused a synergistic induction of apoptosis which was mediated through an increase in caspase activation. The combinational treatment promoted the JNK-Bcl-xL-Bak pathway which transmitted the synergistic effect through the mitochondria-dependent apoptotic pathway. JNK signaling led to Bcl-xL phosphorylation at serine 62, dissociation of Bak from Bcl-xL, oligomerization of Bak, alteration of mitochondrial membrane potential, and subsequent cytochrome *c* release. Overexpression of dominant-negative mutant of Bcl-xL (S62A), but not dominant-positive mutant of Bcl-xL (S62D), suppressed the synergistic death effect. Interestingly, Beclin-1 was dissociated from Bcl-xL and overexpression of dominant-negative mutant of Bcl-xL (S62A), but not dominant-positive mutant of Bcl-xL (S62D), suppressed dissociation of Beclin-1 from Bcl-xL. A combinatorial treatment of mitomycin C, Ad.TRAIL and hyperthermia induced Beclin-1 cleavage, but the Beclin-1 cleavage was abolished in Beclin-1 double mutant (D133A/D146A) knock-in HCT116 cells, suppressing the apoptosis induced by the combination therapy. We believe that this study supports the application of the multimodality approach to colon cancer therapy.

## Introduction

Colorectal cancer is the third leading cause of cancer-related mortality in the world. The main cause of death of patients with colorectal cancer is metastases [1]. In the last decade, the medical control of metastatic colorectal cancer has shown remarkable advances. For example, the addition of oxaliplatin and irinotecan to previously existing 5-fluorouracil (5-FU) and leucovorin-based therapies improved the overall median survival of 12 months [2], and with the addition of biological targeted therapies such as bevacizumab and cetuximab, the overall median survival rose to 20 months [3–8]. However, systemic chemotherapeutic strategies are currently used only as palliatives—they do not improve long-term survival rates. Therefore, new approaches are necessary to improve the overall median survival for metastatic colorectal cancers such as hepatic colorectal metastases and colorectal peritoneal carcinomatosis.

Current standard of care for hepatic colorectal metastases and colorectal peritoneal carcinomatosis is hyperthermic isolated hepatic perfusion (HIHP) therapy and hyperthermic intraperitoneal chemotherapy (HIPEC), respectively [9, 10]. These therapies are combinatorial treatments of chemotherapeutic agents and mild hyperthermia. We hypothesize that a multimodality [chemotherapeutic agent mitomycin C, biologic agent tumor necrosis factor-related apoptosis-inducing ligand (TRAIL/Apo-2L), and mild hyperthermia] approach effectively enhances apoptotic death and improves the current standard of care to treat advanced colorectal metastases. To test the hypothesis, in this study, we focus on the effect of multimodality treatment on cell death in in vitro system.

Chemotherapy is the standard clinical treatment for human cancers. Chemotherapeutics can interact with DNA and activate several intracellular signal pathways including apoptosis [11]. Mitomycin C is an antibiotic which has been used as a chemotherapeutic agent in a variety of carcinogenic solid tumors including colon [12], breast [13], lung [14] and bladder [15]. Mitomycin C causes cross-links in the DNA molecules of the cells, leading to the formation of DNA interstrands and inhibiting DNA replication and transcription [16, 17]. Previous reports suggest that mitomycin C produces a consistent response when used as a single agent [18–21]. However, like many other chemotherapeutic agents, mitomycin C may induce resistance to chemotherapy and may target normal cells, thus causing unexpected side effects at therapeutic doses. On the other hand, since mitomycin C offers a suitably low toxicity and can be easily delivered to an outpatient, it seems a reasonable, cost-effective candidate for combinational therapy of colorectal cancer, like irinotecan [22], capecitabine [23] or 5-FU [24].

TRAIL, a highly promising anticancer agent, is a cytokine of the TNF family which binds to the death receptors DR4 [25–27] and DR5 [28–30]. It has the unique ability to induce apoptosis in many transformed cancer cell lines, but not in normal tissue [31, 32]. Previous studies suggest that TRAIL-induced apoptosis can be enhanced by combinational therapy with several chemotherapeutic agents at non-toxic doses in cancer cells [33–38]. Nonetheless, the translation of TRAIL into the clinic has been confounded by its short half-life, inadequate delivery methods, and TRAIL-resistant cancer cell populations [39]. To solve these limitations, we attempt to develop a secretory TRAIL-armed adenoviral vector.

Hyperthermia has been explored as an anticancer agent for many decades and is often used with HIPEC. We previously reported that hyperthermia has a synergistic effect with TRAIL in causing cytotoxicity through the mitochondria-dependent pathway [40–43]. Several researchers also reported that hyperthermia acts synergistically with ionizing radiation [44–46], and with a number of chemotherapeutic agents [47–49]. We previously reported that hyperthermia triggered down-regulation of c-FLIP_L_ (long form of cellular FLICE-inhibitory protein), an anti-apoptotic molecule, in several colon cancer cells [50]. c-FLIP splice variants (long and short form) bind to FADD and/or caspase 8/10 and inhibit their activation [51–53].

In this study, we observed that a combinatorial treatment of mitomycin C, adenoviral TRAIL and hyperthermia effectively activates the mitochondrial-dependent apoptotic pathway by activating the JNK-Bcl-xL-Bak pathway, increasing Bak oligomerization, facilitating cytochrome *c* release, promoting dissociation of Beclin-1 from Bcl-xL, and increasing Beclin-1 cleavage.

## Materials and methods

### Cell culture

The human colon adenocarcinoma line LS174T was kindly provided by Dr. HA Choudry (University of Pittsburgh Medical Center, Pittsburgh, PA, USA) and LS180 was purchased from the American Type Culture Collection (ATCC, Manassas, VA, USA). The human colorectal carcinoma HCT116 wild type was obtained from Dr. B Vogelstein (Johns Hopkins University) and CX-1 cell line was obtained from Dr. JM Jessup (National Cancer Institute). Cell lines were maintained in Dulbecco’s Modified Eagle Medium (DMEM, Gibco, Gaithersburg, MD, USA), McCoy’s 5A or RPMI 1640 supplemented with 10 % fetal bovine serum (FBS, Atlanta Biological, Flowery Branch, GA, USA), 100 U/mL penicillin and 100 µg/mL streptomycin (Gibco) in a 5 % CO_2_ incubator. These adherent cells, except for LS180, were subcultured every 3–4 days by treatment with a trypsin–EDTA solution (Gibco). The LS180 cell line was cultured by following a procedure of ATCC subculturing.

### Reagents and antibodies

Mitomycin C and anti-TRAIL antibody were obtained from Santa Cruz Biotechnology (Dallas, TX, USA). Anti-PARP, anti-caspase 8/9/3, anti-cleaved caspase 9/3, anti-phosphorylated (Thr183/Tyr185) JNK, anti-JNK, anti-Bcl-xL, anti-COX-IV and anti-human influenza hemagglutinin (HA) antibody were purchased from Cell Signaling (Danvers, MA, USA). Anti-phosphorylated Bcl-xL (S62) antibody was purchased from Millipore (Billerica, MA, USA) and anti-cytochrome *c* antibody and anti-Beclin-1 were from BD Pharmigen (San Jose, CA, USA). Anti-actin antibody and other chemicals were purchased from Sigma (St. Louis, MO, USA).

### Shuttle vector construction

pFETZ was kindly provided by Dr. Y He (Immunotherapy Center, Medical College of Georgia, GA, USA) [54]. This vector contains cDNA for the extracellular domain of Flt3L, a ligand for Flt3 tyrosine kinase receptor (a.a. 1–81), fused to a leucine zipper domain and the extracellular domain of TRAIL (a.a. 95–281) and expresses a secretable form of TRAIL fusion protein [55]. pAdlox-FETZ was made by inserting the SalI/BamHI fragment from pFETZ into SalI/BamHI-cut pAdlox shuttle vector. The complete shuttle vector was co-transfected into CRE8 cells with ψ5 viral genomic DNA for homologous recombination as described below.

### Adenoviral vector construction

Recombinant adenovirus was constructed by employing the Cre-lox recombination system [56]. The selective cell line CRE8 has a β-actin-based expression cassette driving a Cre recombinase gene with an N-terminal nuclear localization signal stably integrated into 293 cells. Transfections were done by using Lipofectamine reagent (Invitrogen, Carlsbad, CA, USA). Cells were split into T25-flasks 1 day before transfection. For the production of recombinant adenovirus, 2 µg of Adlox/FETZ and 2 µg of ψ5 viral genomic DNA were co-transfected into CRE8 cells in the presence of caspase inhibitor z-VAD-fmk (20 μM). The recombinant adenoviruses were generated by intramolecular homologous recombination between the shuttle vector and ψ5 viral DNA. The new virus had an intact packaging site and carried a recombinant gene. Plaques were harvested, analyzed and purified.

### Drug treatment

Drug treatments were accomplished by aspirating the medium and replacing it with new medium containing drugs.

### Hyperthermia treatment

Cells cultured in 35 mm or 100 mm dishes were sealed with parafilm and were placed a circulating water bath (Heto, Thomas Scientific, Denmark), which was maintained within 0.02 °C of the desired temperature.

### Survival assay

One or 2 days prior to the experiment, human colorectal carcinoma LS174T cells were plated into six well plates. For trypan blue exclusion assay, trypsinized cells were pelleted and resuspended in 0.2 mL of medium, 0.5 mL of 0.4 % trypan blue solution, and 0.3 mL of phosphate-buffered saline solution (PBS). The samples were mixed thoroughly, incubated at room temperature for 15 min, and examined by automated cell counter (LUNA, Logos Biosystems, Annandale, VA, USA). At least 300 cells were counted for each survival determination.

### Protein extracts and polyacrylamide gel electrophoresis (PAGE)

Cells were scraped with 1 × Laemmli lysis buffer [including 2.4 M glycerol, 0.14 M Tris (pH 6.8), 0.21 M SDS, and 0.3 mM bromophenol blue] and boiled for 3 min. Protein concentrations were measured with BCA protein assay reagent (Pierce, Rockford, IL, USA). The samples were diluted with 1 × lysis buffer containing 20 mM dithiothreitol (DTT), and an equal amount of protein was loaded on 10–15 % SDS-polyacrylamide gels. SDS-PAGE analysis was performed using a Hoefer gel apparatus.

### Immunoblot analysis

Proteins were separated by SDS-PAGE, electrophoretically transferred to nitrocellulose membranes and blocked with 5 % skim milk in TBS-Tween 20 (0.05 %, v/v) for 30 min. The membrane was incubated with primary antibodies for 2 h at room temperature or overnight at 4 °C. Horseradish peroxidase-conjugated anti-rabbit or anti-mouse IgG was used as the secondary antibody. Immunoreactive protein was visualized by the enhanced chemi-luminescence protocol (ECL, Amersham, Arlington Heights, IL, USA).

### Annexin V binding

The translocation of phosphatidylserine, one of the markers of apoptosis, from the inner to the outer plasma membrane was detected by binding of allophycocyanin (APC)-conjugated Annexin V. LS174T cells were plated into plates, treated with drugs for 24 h and stained with mouse anti-human Annexin V antibody and propidium iodide (PI). The staining was terminated and cells were immediately analyzed by flow cytometry.

### JC-1 mitochondrial membrane potential assay

After drug treatment, cells were stained using JC-1 mitochondrial membrane potential detection kit (Invitrogen) for 10 min and analyzed by flow cytometry. Fluorescence intensity was measured with the Accuri C6 flow cytometer (Accuri Cytometers, Inc., San Jose, CA, USA). Results were analyzed with VenturiOne software (Applied Cytometry, Inc., Plano, TX, USA).

### Cytochrome c release assay

To determine the release of cytochrome *c* from the mitochondria, LS174T cells growing in 100 mm dishes were used. After drug treatment, mitochondria and cytosolic fractions were prepared from treated cells by using a Mitochondrial Fractionation Kit (Active Motif, Carlsbad, CA, USA) following company instructions and reagents included in the kit. Cytosolic fractions were subjected to SDS-PAGE and analyzed by immunoblotting using anti-cytochrome *c* antibody. Equal loading of the mitochondrial pellets was confirmed with anti-COX IV antibody.

### Bak oligomerization

After drug treatment, cells were collected and resuspended in homogenization buffer. The cell suspension was homogenized several times and centrifuged at 1,000×*g* for 10 min at 4 °C to obtain nuclear pellets. Supernatant was transferred to a new 1.5 mL tube and spun at 10,000×*g* for 15 min at 4 °C to pellet the mitochondria. Isolated mitochondrial fractions and cytosolic fractions were cross-linked with 1 mmol/L dithiobis (Pierce) for 1 h at room temperature. After cross-linking, the mitochondria were pelleted and samples were subjected to SDS-PAGE under nondenaturing conditions followed by immunoblotting for Bak.

### Stable transfection

Cells stably overexpressing HA-Bcl-xL wild-type (WT) or mutant types were prepared by transfecting CX-1 cells with human Bcl-xL tagged with HA epitope in pCDNA3.1 vector: HA-Bcl-xL WT, HA-Bcl-xL S62A (mutation of serine 62 to alanine), and HA-Bcl-xL S62D (mutation of serine 62 to aspartate; a kind gift from Dr. TC Chambers, University of Arkansas). Cells were maintained in 500 µg/mL G418.

### Immunoprecipitation

Briefly, cells were pelleted and lysed by CHAPS buffer with protease inhibitor cocktail. Cell lysates were completely disrupted by repeated aspiration through a 27 gauge needle. Cell debris was removed and protein concentration was determined by BCA Protein Assay Reagent. For immunoprecipitation, 500 µg of lysate was incubated with 1 µg of rabbit anti-Bcl-xL/anti-HA antibody or rabbit IgG at 4 °C overnight, followed by the addition of protein G-agarose beads and rotation at 4 °C for 4 h. Immunoprecipitates were collected by centrifugation and the pellet was dissolved in electrophoresis sample buffer for heat denaturation. The immune complexes were subjected to SDS-PAGE followed by immunoblot analysis.

### Statistical analysis

Statistical analysis was carried out using GraphPad InStat 3 software (GraphPad Software, Inc., San Diego, CA, USA). Significance was set at values of *(*p* < 0.05), **(*p* < 0.01), or ***(*p* < 0.001).

## Results

### Characterization of a secretory TRAIL-armed adenoviral vector

**Fig. 1:**
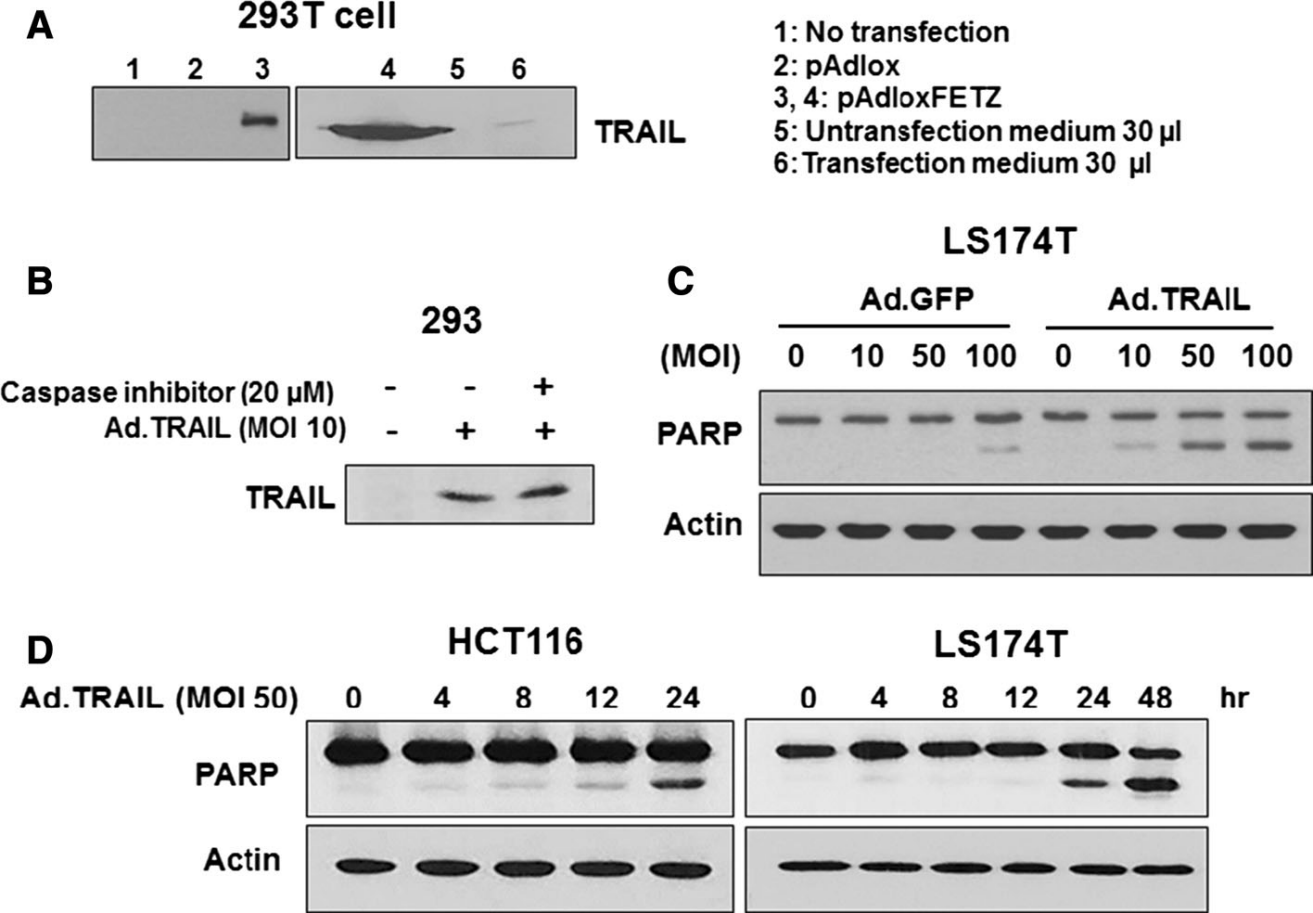
Production of secretory TRAIL and its cytotoxicity. **a** 293 cells were non-transfected (lane 1), or transfected with 4 µg pAdlox empty vector (lane 2) or pAdlox-FETZ vector (lane 3, 4) and then incubated for 48 h. After incubation, 30 µg of cell lysate protein (lane 1, 2, 3, and 4) or 30 µL of cell culture medium (lane 5, 6) were immunoblotted with anti-TRAIL antibody. **b** 293 cells were infected with Ad.TRAIL (MOI 10) in the presence/absence of caspase inhibitor (20 µM). Cell lysates were prepared in lysis buffer and immunoblotted with anti-TRAIL. **c** LS174T cells were infected by various MOI of Ad.TRAIL or Ad.GFP for 24 h and harvested. Cell lysates were immunoblotted with anti-PARP antibody. **d** HCT116 and LS174T cells were infected with Ad.TRAIL (MOI 50) and harvested at various time points. Cell lysates were immunoblotted with anti-PARP antibody. Actin was used as an internal standard

We constructed pAdlox-FETZ shuttle vector which has a CMV promoter-driven secretory human Flt3 ligand-isoleucine zipper-human TRAIL fusion protein gene. This plasmid encoded the secretable form of chimeric TRAIL proteins which were detected in Fig. 1a. Next, we constructed a secretory TRAIL-armed adenoviral vector (Ad.TRAIL) and characterized the vector. Data from Fig. 1b shows an expression of TRAIL in Ad.TRAIL infected 293 cells in the absence/presence of caspase inhibitor z-VAD-fmk, which was added to prevent TRAIL-induced apoptosis. Unlike most cancer cell lines, immortalized normal human embryonic kidney 293 cells were resistant to TRAIL. To examine an apoptotic capacity of Ad.TRAIL, human colon adenocarcinoma LS174T cells were infected with various multiplicity of infection (MOI) of Ad.TRAIL and its control adenoviral construct encoding green fluorescent protein (Ad.GFP). As shown in Fig. 1c, Ad.TRAIL induced PARP cleavage, the hallmark feature of apoptosis. TRAIL-induced PARP cleavage was dependent upon MOI. Unlike Ad.TRAIL, Ad.GFP induced minimal apoptosis only at MOI of 100. Next, we investigated the kinetics of apoptosis after Ad.TRAIL infection in human colorectal carcinoma HCT116 and LS174T cells. Figure 1d shows that PARP cleavage occurred 24 h after infection in both cell lines.

### Synergistic induction of apoptosis by Ad.TRAIL in combination with mitomycin C and hyperthermia

**Fig. 2:**
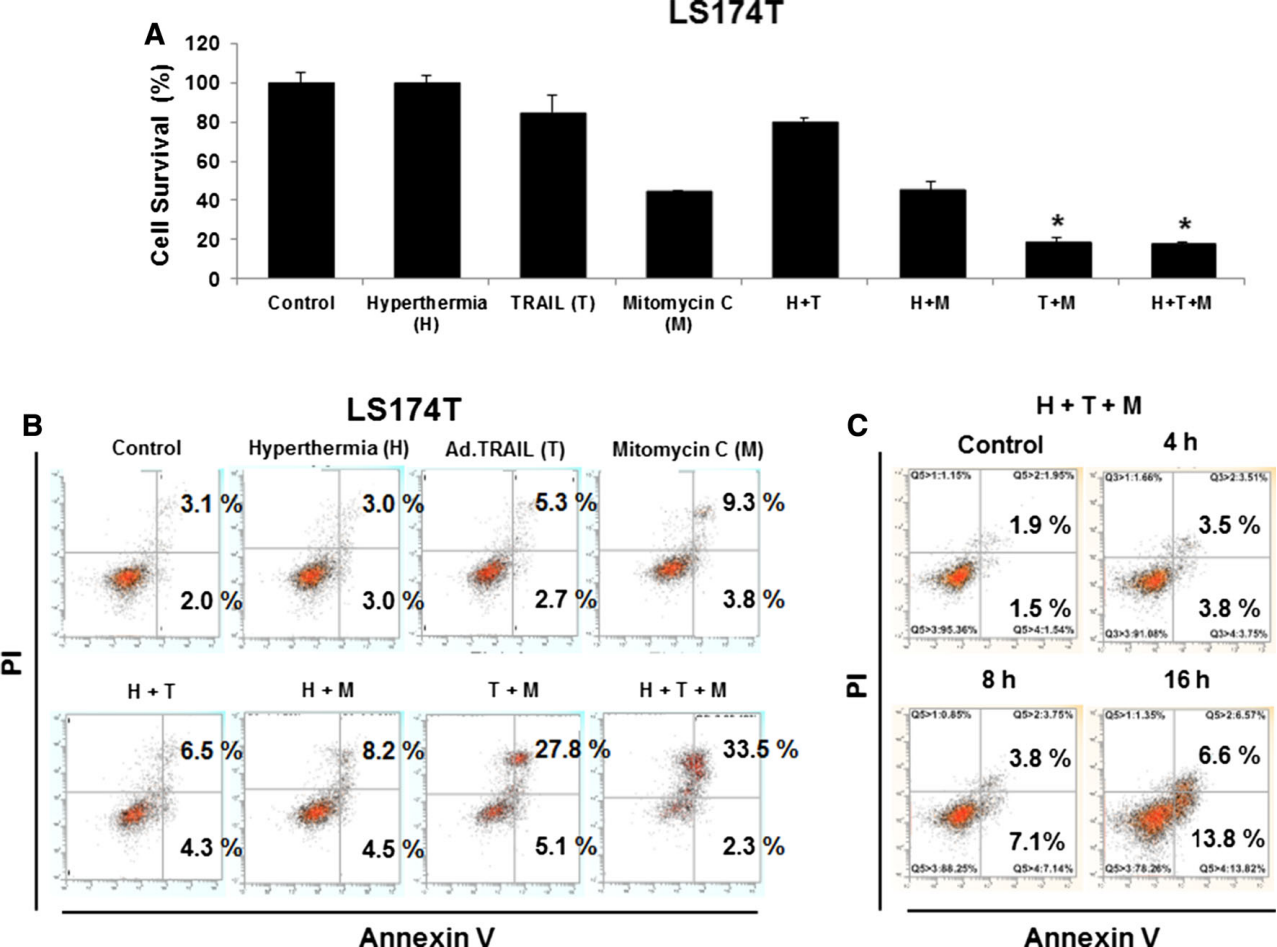
Synergistic induction of cytotoxicity by treatment with Ad.TRAIL in combination with mitomycin C and hyperthermia in LS174T cells. LS174T cells were treated with Ad.TRAIL (MOI 25) or/and mitomycin C (5 µg/mL) for 24 h and exposed to normothermia (37 °C) or hyperthermia (42 °C) for 1 h, and then incubated for 3 h at 37 °C. **a** Cell survival was analyzed by the trypan blue dye exclusion assay. **b** Cells were stained with fluorescein isothiocyanate (FITC)-Annexin V and propidium iodide (PI). **c** Cells were treated with Ad.TRAIL (MOI 25) and mitomycin C (5 µg/mL) for 4, 8, and 16 h and exposed to hyperthermia (42 °C) for 1 h, and then incubated for 3 h at 37 °C. Apoptosis was detected by the flow-cytometric assay

**Fig. 3:**
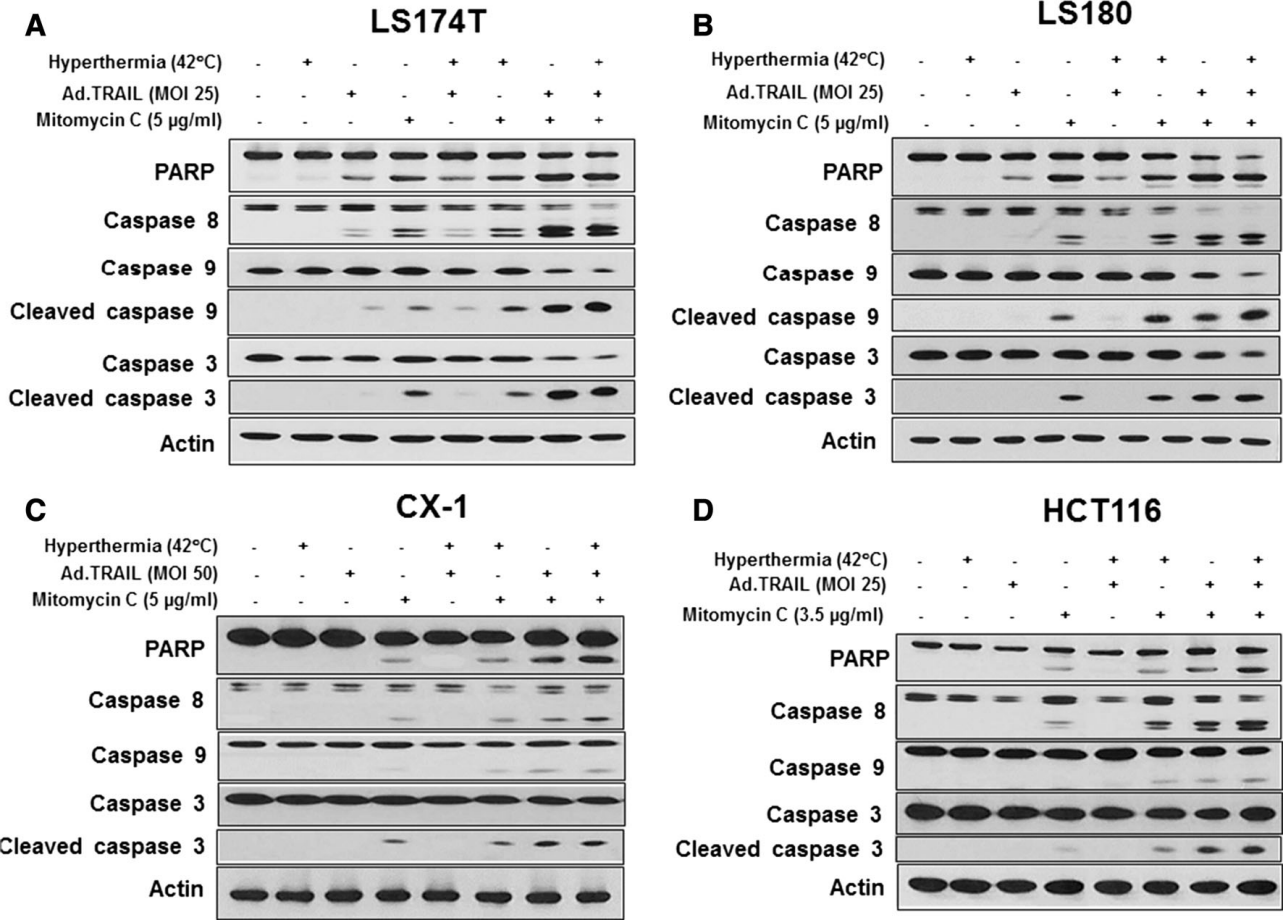
Synergistic induction of apoptosis by treatment with Ad.TRAIL in combination with mitomycin C and hyperthermia in colon cancer cells. LS174T (**a**), LS180 (**b**), CX-1 (**c**) and HCT116 (**d**) cells were treated with Ad.TRAIL (MOI 25 or 50) or/and mitomycin C (3.5 or 5 µg/mL) for 24 h and exposed to normothermia (37 °C) or hyperthermia (42 °C) for 1 h, and then incubated for 3 h at 37 °C. After treatment, the cleavage of caspase 8/9/3 or PARP was detected by immunoblotting. Actin was used to confirm the equal amount of proteins loaded in each lane

To investigate the effect on cell viability of the application of Ad.TRAIL in combination with mitomycin C and hyperthermia, LS174T cells were infected with Ad.TRAIL (MOI 25) and treated with 5 µg/mL mitomycin C for 24 h. After treatment, the cells were heated at 42 °C for 1 h and incubated at 37 °C for 3 h and then cell viability was determined by trypan blue dye exclusion assay. As shown in Fig. 2a, synergistic cytotoxic effect was observed in Ad.TRAIL + mitomycin C or Ad.TRAIL + mitomycin C + hyperthermia compared with any single treatment (*p* < 0.05). We further investigated whether the combinatorial treatment-induced cytotoxicity was associated with apoptosis. Data from the flow cytometry assay demonstrate that the combinatorial treatment-induced cytotoxicity was associated with apoptosis (Figs. 2b, c); apoptotic death cells were observed in the lower right quadrant (early apoptotic death) and the upper right quadrant (late apoptotic death) of the plots. The results from Figs. 2b, c clearly demonstrate that apoptosis was time-dependent during combined treatment. Figures 2b, c clearly show that treatment with Ad.TRAIL in combination with mitomycin C and hyperthermia enhanced synergistic induction of apoptotic death. These synergistic effects were due to an increased activation (cleavage) of caspase 8/9/3 and thus, the hallmark feature of apoptosis, PARP cleavage (Fig. 3a). Similar results were observed in LS180, CX-1 and HCT116 cell lines (Figs. 3b, c, d). These results indicate that synergistic induction of apoptosis by combinatorial treatment with mitomycin C/Ad.TRAIL/hyperthermia is mediated through an increase in caspase activation.

### Role of the JNK-Bcl-xL-Bak pathway in the combinatorial treatment-induced apoptosis

**Fig. 4:**
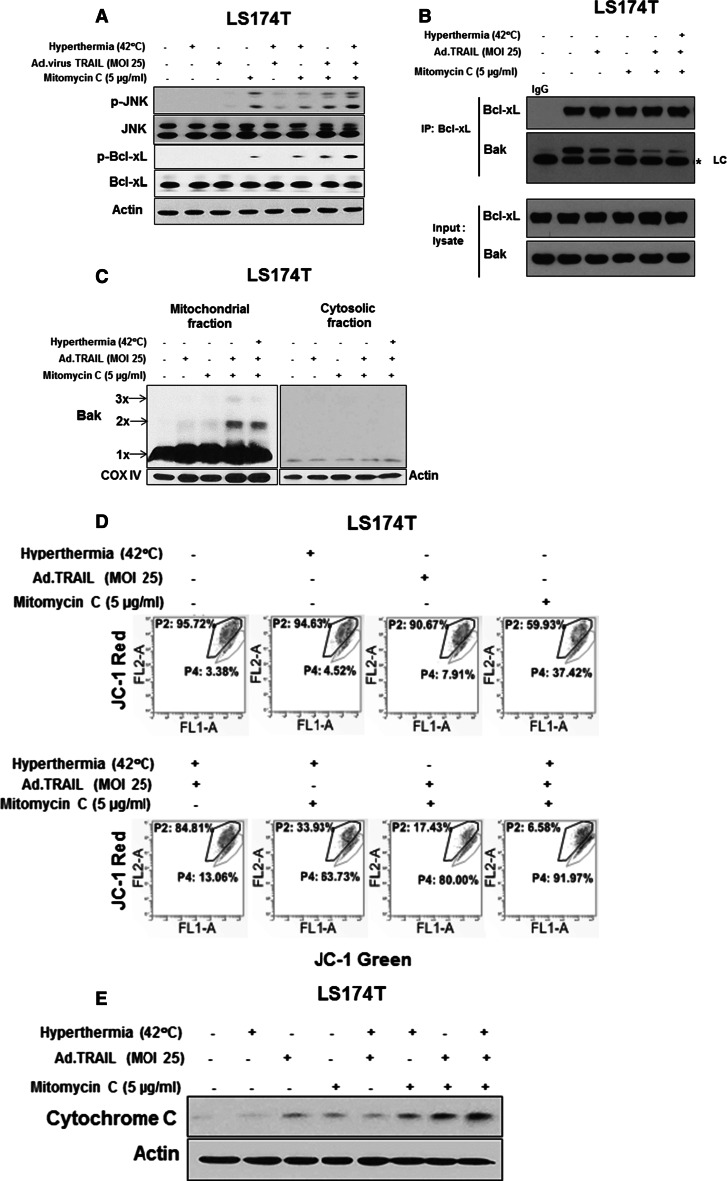
Ad.TRAIL in combination with mitomycin C and hyperthermia-induced activation of the JNK-Bcl-xL pathway, Bak oligomerization, mitochondrial membrane potential change and cytochrome *c* release. LS174T cells were treated with Ad.TRAIL (MOI 25) or/and mitomycin C (5 µg/mL) for 24 h and exposed to normothermia (37 °C) or hyperthermia (42 °C) for 1 h, and then incubated for 3 h at 37 °C. **a** After treatment, cell lysates containing equal amounts of protein were separated by SDS-PAGE and immunoblotted with anti-phospho-JNK, anti-JNK, anti-phospho Bcl-xL or anti-Bcl-xL antibody. **b** Cell lysates were immunoprecipitated with anti-Bcl-xL antibody or IgG and immunoblotted with anti-Bak antibody. The presence of Bcl-xL and Bak in the lysates was examined. Asterisk (*) is IgG light chain (LC). **c** Mitochondrial and cytosolic fractions were isolated and were cross-linked with 1 M DSP (dithiobis, succinimidyl propionate) for 30 min and then subjected to immunoblotting with anti-Bak antibody. Bak monomer (×1) and multimer (×2, ×3) are indicated. COX IV and actin were shown as an internal standard of mitochondrial fraction and cytosolic fraction, respectively. **d** Cells were stained with JC-1 and then analyzed by flow cytometry. **e** Cytochrome *c* release into cytosol was determined by immunoblotting for cytochrome *c* in the cytosolic fraction. Actin was used to confirm the equal amount of proteins loaded

We previously reported that the JNK-Bcl-xL pathway plays an important role in the synergistic effect on apoptosis of treatment with multiple cytotoxic agents [43, 57]. This possibility was examined during treatment with mitomycin C/Ad.TRAIL/hyperthermia. Phosphorylation of JNK and Bcl-xL was observed during treatment with mitomycin C/Ad.TRAIL/hyperthermia in LS174T cells (Fig. 4a). Even with treatment with only mitomycin C, phosphorylation of JNK and Bcl-xL was detected. Moreover, an increase in phosphorylation was observed during combinatorial therapy. Data from immunoprecipitation assay show that the combinatorial treatment induced the dissociation of Bak from Bcl-xL (Fig. 4b). We previously reported that phosphorylation of Bcl-xL alters the interactions between Bcl-xL and Bax and then leads to Bax oligomerization [43]. Since the presence of Bax was not detected in LS174T cells (data not shown), we examined Bak oligomerization. Bak oligomerization occurred during treatment with Ad.TRAIL in combination with mitomycin C with/without hyperthermia (Fig. 4c). Oligomerized Bak may bind to the mitochondria, altering mitochondrial membrane potential (Fig. 4d) and causing a cytochrome c release (Fig. 4e).

### Alteration of interactions between Bcl-xL and Beclin-1 and cleavage of Beclin-1 during the combinatorial treatment

**Fig. 5:**
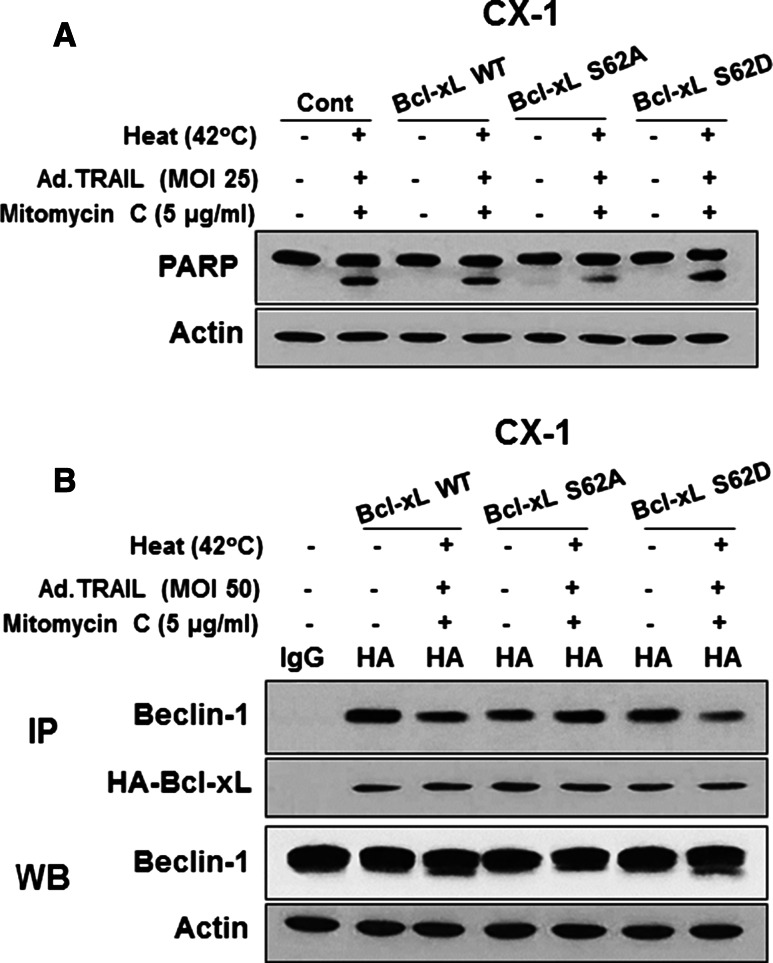
Role of Bcl-xL in apoptosis. CX-1 cells were stably transfected with HA-Bcl-xL WT, HA-Bcl-xL S62A or HA-Bcl-xL S62D plasmid and then treated with Ad.TRAIL (MOI 25) and/or mitomycin C (5 µg/mL) for 24 h and exposed to normothermia (37 °C) or hyperthermia (42 °C) for 1 h, and then incubated for 3 h at 37 °C. **a** After treatment, lysates containing equal amounts of protein were separated by SDS-PAGE and PARP cleavage was detected by immunoblotting. Actin was used as an internal standard. **b** Cell lysates were immunoprecipitated with anti-HA antibody or mock antibody (IgG) and immunoblotted with anti-Beclin-1 or anti-HA antibody (*upper panels*). The presence of Beclin-1 and actin in the lysates was examined (*lower panels*)

**Fig. 6:**
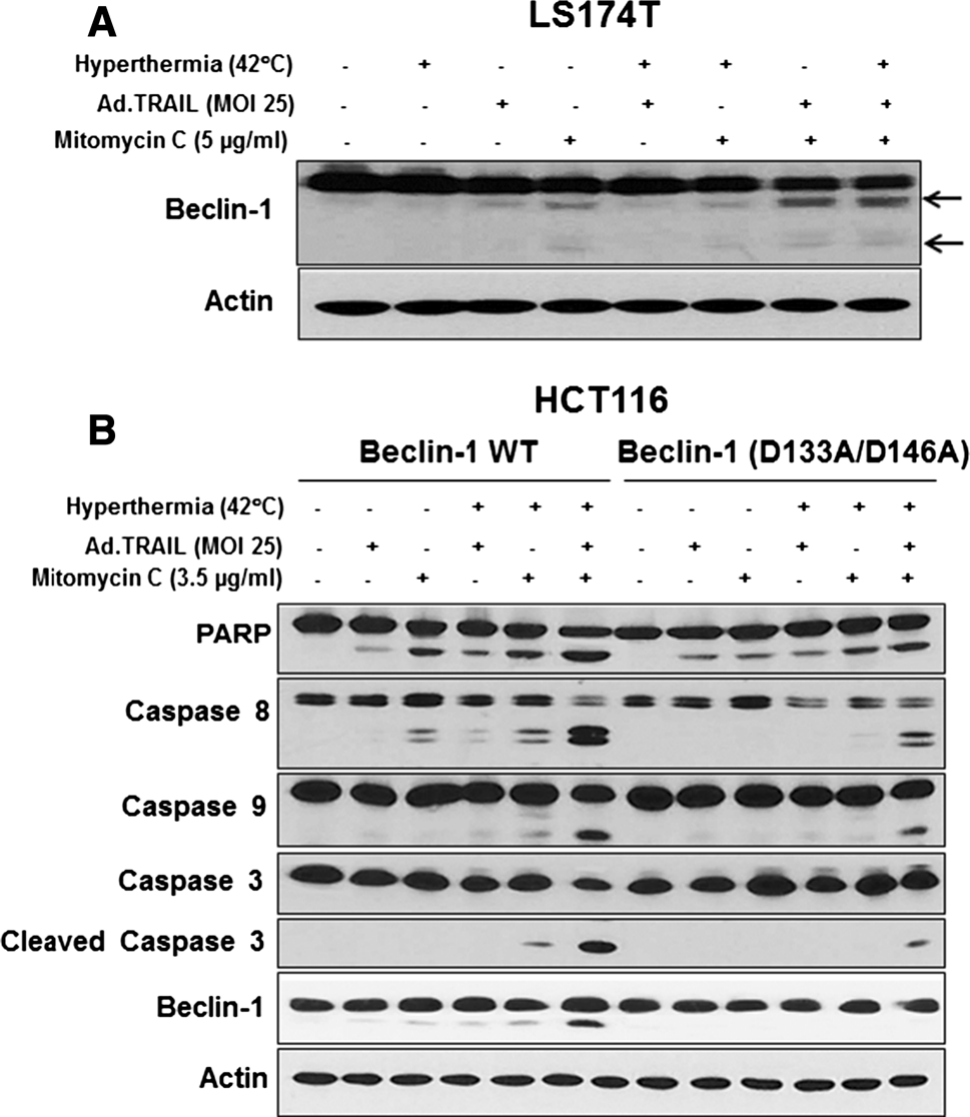
Role of Beclin-1 in Ad.TRAIL in combination with mitomycin C and hyperthermia-induced cell death. LS174T, HCT116 and HCT116 Beclin-1 knock-in (D133A/D146A) cells were treated with Ad.TRAIL (MOI 25) or/and mitomycin C (3.5 or 5 µg/mL) for 24 h and exposed to normothermia (37 °C) or hyperthermia (42 °C) for 1 h, and then incubated for 3 h at 37 °C. **a** LS174T cell lysates were immunoblotted by Beclin-1 antibody. *Arrows* indicate cleaved Beclin-1. **b** Parental HCT116 (HCT116 WT) and HCT116 D133A/D146A cell lysates were immunoblotted with anti-PARP, anti-caspase 8/9/3, or anti-Beclin-1 antibody. Actin was shown as an internal standard

To examine whether phosphorylation of the S62 residue on Bcl-xL is important for apoptosis, CX-1 cells were stably transfected with plasmid containing HA-Bcl-xL WT, phosphor-defective HA-Bcl-xL S62A or phosphor-mimic HA-Bcl-xL S62D. Figure 5a shows that HA-Bcl-xL S62A, but not HA-Bcl-xL WT and HA-Bcl-xL S62D, inhibited apoptosis. These results suggest that the phosphorylation of Bcl-xL plays an important role in the combinatorial treatment-induced apoptosis. We previously reported that phosphorylation of Bcl-xL affects interaction between Bcl-xL and Beclin-1, causing dissociation of Beclin-1 from Bcl-xL [57]. Data from immunoprecipitation assay show that overexpression of dominant-negative mutant type of Bcl-xL S62A, but not wild type Bcl-xL WT or dominant-positive mutant type of Bcl-xL S62D, suppressed the dissociation of Beclin-1 from Bcl-xL (Fig. 5b). Several researchers reported that Beclin-1 has two cleavage sites at D133 and D146 residues [58] and that Beclin-1 is cleaved by caspase 8, and C-terminal fragment of Beclin-1 localizes at the mitochondria and then induces cytochrome *c* release [58, 59]. Figure 6a shows that the combinatorial treatment enhanced Beclin-1 cleavage. On Fig. 6b, data from Beclin-1 double mutant (D133A/D146A) knock-in HCT116 cells show suppression of cleavage of PARP and caspase 8/9/3 (apoptosis). Beclin-1, a mammalian homolog of yeast autophagy-related protein 6 (Atg6), functions in autophagy by initiating autophagosome formation [60]. However, it has been suggested that crosstalk between apoptosis and autophagy is associated with caspase-mediated cleavage of Beclin-1 which destroys its pro-autophagic activity and can then amplify mitrochondrion-mediated apoptosis through the cleaved C-terminal fragment [58]. Our data suggest that the combinatorial treatment-induced synergistic apoptotic death is mediated through Beclin-1 cleavage.

## Discussion

Several conclusions can be drawn upon consideration of the data presented here. First, a combinatorial treatment of Ad.TRAIL, mitomycin C and hyperthermia synergistically induces apoptosis. Second, the JNK-Bcl-xL-Bak pathway plays an important role in the apoptosis through activating the mitochondria-dependent pathway. Third, a combinatorial treatment of Ad.TRAIL, mitomycin C and hyperthermia induces dissociation of Beclin-1 from Bcl-xL and promotes Beclin-1 cleavage which results in enhancement of apoptosis.

The DNA damage surveillance network may orchestrate cellular responses to mitomycin C-induced DNA damage through the recruitment of DNA damage sensing molecules, transducer and effector proteins [61]. Ataxia telangiectasia mutated (ATM) and Nijmegen breakage syndrome (NBS1) cooperatively sense DNA damage and post-translationally modify transducers such as BRCA1 (early-onset familial breast and ovarian cancer), MDC1 (mediator of DNA damage checkpoint 1), 53BP1 (p53-binding protein 1), and c-Abl [62, 63]. These modified transducer proteins amplify and transduce the signals to downstream effectors such as H2AX, p53, Chk2 and JNK [64–72]. It is possible that mitomycin C-activated JNK is mediated through the ATM-c-Abl signal transduction pathway. This possibility needs to be further investigated.

We previously reported that Bcl-xL undergoes phosphorylation in response to treatment with oxaliplatin, TRAIL/mapatumumab and hyperthermia [42, 43, 57, 73–75]. Bcl-xL phosphorylation requires activated JNK, which can recognize a proline residue on the carboxyl side of the phospho-acceptor [76]. Some studies reported Bcl-xL phosphorylation to occur on serine 62, while others reported it to occur on threonines 47 and 115 [77, 78]. This study with site-directed mutagenesis at Ser-62 showed that cells expressing a validated phospho-defective Bcl-xL mutant are resistant to the combinatorial treatment of Ad.TRAIL, mitomycin C and hyperthermia-induced apoptosis, whereas cells expressing a phospho-mimic Bcl-xL are sensitive to the combinatorial treatment-induced apoptosis, indicating that phosphorylation at Ser-62 is a key regulatory mechanism for antagonizing anti-apoptotic function in the combinatorial treatment.

Previous studies have shown that JNK1-mediated phosphorylation of Bcl-2 at residues T69, S70, and S87 is required for dissociation of Bcl-2 from Beclin-1 and autophagy activation [79]. Unlike Bcl-2, data from Fig. 5b suggests that for Bcl-xL, phosphorylation only at residue S62 may be sufficient for Bcl-xL dissociation from Beclin-1. It was reported that D133 and D146 of Beclin-1 are cleaved by caspase 8 during apoptosis [58, 59]. Caspase 8-mediated cleavage of Beclin-1 inactivates Beclin-1-induced autophagy and enhances apoptosis by promoting the release of proapoptotic factors from mitochondria [58, 59]. Studies with the caspase 8-resistant Beclin-1 knockin cell line clearly demonstrate that the combinatorial treatment-induced Beclin-1 cleavage and apoptosis were reduced (Fig. 6b), and Beclin-1’s autophagy-promoting function was restored (data not shown) in Beclin-1 KI HCT116 cells.

We reported that hyperthermia has a synergistic effect with TRAIL in causing apoptosis [40–42]. We also reported that hyperthermia triggers down-regulation of c-FLIP_L_ (long form of cellular FLICE-inhibitory protein), an anti-apoptotic molecule, through ubquitination in several colon cancer cell lines [50]. It has been found that c-FLIP splice variants (long and short form) bind to FADD and/or caspase 8/10 and inhibit their activation [51–53]. Thus, down-regulation of c-FLIP_L_ is probably responsible for hyperthermia-enhanced TRAIL cytotoxicity. Interestingly, long-term pretreatment with TRAIL by expressing TRAIL from Ad.TRAIL showed a minimal synergistic effect with hyperthermia, even though down-regulation of c-FLIP_L_ was observed (data not shown). This may be due to the activation of TRAIL-associated death signals prior to hyperthermia. This possibility needs to be further studied.

Our data illustrate that a combinatorial treatment of Ad.TRAIL, mitomycin C and mild hyperthermia synergistically induces apoptosis and effectively activates the mitochondria-dependent apoptotic pathway by activating the JNK-Bcl-xL-Bak pathway. Moreover, our data suggest that Beclin-1 is dissociated from phosphorylated Bcl-xL and cleaved during treatment with Ad.TRAIL, mitomycin C and hyperthermia. The cleavage of Beclin-1 promoted the combinatorial treatment-induced apoptotic death. The studies presented here further elucidate a crosstalk between the JNK-Bcl-xL-Bak pathway and the Bcl-xL/Beclin-1-mediated pathway during treatment with Ad.TRAIL, mitomycin C and hyperthermia. A greater understanding of these interactions may be critical for enhancing the combinatorial treatment.

## Abbreviations

Ad.TRAIL: TRAIL-armed adenovirus
APC: Allophycocyanin
ATM: Ataxia telangiectasia mutated
53BP1: p53-binding protein 1
BAX: Bcl-2–associated X protein
Bcl-xL: B-cell lymphoma-extra large
BRCA1: Early-onset familial breast and ovarian cancer
c-FLIP_L_: Long form of cellular FLICE-inhibitory protein
Chk2: Checkpoint kinase 2
COX: Cyclooxygenase
CRE8: Cre-expressing 293 cells
DMEM: Dulbecco’s modified eagle medium
DR4: Death receptor 4
DR5: Death receptor 5
DTT: Dithiothreitol
EDTA: Ethylenediaminetetraacetic acid
5-FU: 5-fluorouracil
FADD: Fas-Associated protein with Death Domain
FBS: Fetal bovine serum
FLICE: FADD-like interleukin-1 beta-converting enzyme
Flt3: fms-related tyrosine kinase 3
Flt3L: Ligand for Flt3
HA: Hemagglutinin
HIPEC: Hyperthermic intraperitoneal chemotherapy
JNK: c-Jun NH2-terminal kinase
KI: Knock-in
MDC1: Mediator of DNA damage checkpoint 1
NBS1: Nijmegen breakage syndrome
PARP: Poly (ADP-ribose) polymerase
PBS: Phosphate-buffered saline solution
PI: Propidium iodide
RPMI: Roswell Park Memorial Institute medium
SDS: Sodium dodecyl sulfate
PAGE: Polyacrylamide gel electrophoresis
BH3: Bcl-2 homology domain 3
TNF: Tumor necrosis factor
TRAIL: Tumor necrosis factor-related apoptosis-inducing ligand
WT: Wild-type
